# Development of a promising PPAR signaling pathway-related prognostic prediction model for hepatocellular carcinoma

**DOI:** 10.1038/s41598-024-55086-6

**Published:** 2024-02-28

**Authors:** Qingmiao Shi, Yifan Zeng, Chen Xue, Qingfei Chu, Xin Yuan, Lanjuan Li

**Affiliations:** State Key Laboratory for Diagnosis and Treatment of Infectious Diseases, National Clinical Research Center for Infectious Diseases, National Medical Center for Infectious Diseases, Collaborative Innovation Center for Diagnosis and Treatment of Infectious Diseases, The First Affiliated Hospital, Zhejiang University School of Medicine, 79 Qingchun Rd., Hangzhou City, 310003 China

**Keywords:** Hepatocellular carcinoma, PPAR signaling pathway, Prognostic prediction model, Prognostic markers, Cancer microenvironment, Cancer models, Machine learning

## Abstract

The peroxisome proliferator-activated receptor (PPAR) signaling pathway plays a crucial role in systemic cell metabolism, energy homeostasis and immune response inhibition. However, its significance in hepatocellular carcinoma (HCC) has not been well documented. In our study, based on the RNA sequencing data of HCC, consensus clustering analyses were performed to identify PPAR signaling pathway-related molecular subtypes, each of which displaying varying survival probabilities and immune infiltration status. Following, a prognostic prediction model of HCC was developed by using the random survival forest method and Cox regression analysis. Significant difference in survival outcome, immune landscape, drug sensitivity and pathological features were observed between patients with different prognosis. Additionally, decision tree and nomogram models were adopted to optimize the prognostic prediction model. Furthermore, the robustness of the model was verified through single-cell RNA-sequencing data. Collectively, this study systematically elucidated that the PPAR signaling pathway-related prognostic model has good predictive efficacy for patients with HCC. These findings provide valuable insights for further research on personalized treatment approaches for HCC.

## Introduction

Hepatocellular carcinoma (HCC) is an adverse outcome in patients with cirrhosis, particularly prevalent in Asia and posing a substantial disease burden^1,2^. Despite advances in treatment, its high recurrence rate makes HCC a major challenge in clinical decision-making. Peroxisome proliferator-activated receptors (PPARs), including PPARα, PPARδ and PPARγ, are ligand-activated transcription factors of nuclear hormone receptor superfamily^3^. PPARs play a vital role in systemic cell metabolism, energy homeostasis and immune response inhibition^4–6^. Over the last decade, PPARs have been extensively evaluated in basic and clinical research, serving as drug targets for various human diseases. These include diabetes mellitus type 2, hyperlipidemia, nonalcoholic fatty liver disease (NAFLD), and the prevention of inflammatory processes such as primary biliary cholangitis (PBC)-induced liver fibrosis^7,8^. Recently, cumulative evidence have suggested the potential effectiveness of PPAR agonist, such as bezafibrate (PPARα agonist), saroglitazar (PPARα/γ agonist), seladelpar (PPARδ agonist) and elafibranor (PPARα/δ agonist), for the treatment of cholestatic liver disease^9,10^.

Cumulative evidence have suggested that PPAR signaling is closely associated with bile acid metabolism, gut microbiota and hepatocyte proliferation^11–13^. For instance, Guomin et al. reported that PPARα promoted liver regeneration after partial hepatectomy (PHx) in mice^14^. Additionally, multiple studies indicated that PPAR signaling pathway might participated in the pathogenesis of HCC. Gomez et al. demonstrated that PPARγ-mediated signaling pathway in rats is involved in the prevention of HCC through pirfenidone^15^. Zhewen et al. revealed that relationship between PPAR-γ and tumour microenvironment (TME)-related immunosuppression, wherein the drug resistance of immune checkpoint inhibitors (ICIs) in HCC is impacted^16^. A cellular-level study revealed that simvastatin improved sorafenib resistance in HCC through the HIF-1α/PPAR-γ/PKM2 pathway^17^. Similarly, natural and synthetic PPAR agonists have displayed enormous potential in the treatment of HCC^18^. However, the bidirectional mechanism of the PPAR pathway in tumorigenicity remains inadequately explored.

Considering the serious health threat posed by HCC, it is vital to develop new and effective prognostic models. A study based on proteomics and bioinformatics suggested that Acyl-CoA oxidase 2 improved the outcome of patients with HCC through the PPARα pathway, making it a promising prognostic marker^19^. In this study, we established a new algorithm based on PPAR signaling pathway-related genes, aiming to predict the outcomes of patients with HCC. Our findings may provide new strategies for clinical management and prognostic assessment of HCC.

## Results

### Gene expression and mutation analysis of PPAR signaling pathway-related genes in HCC

**Figure 1:**
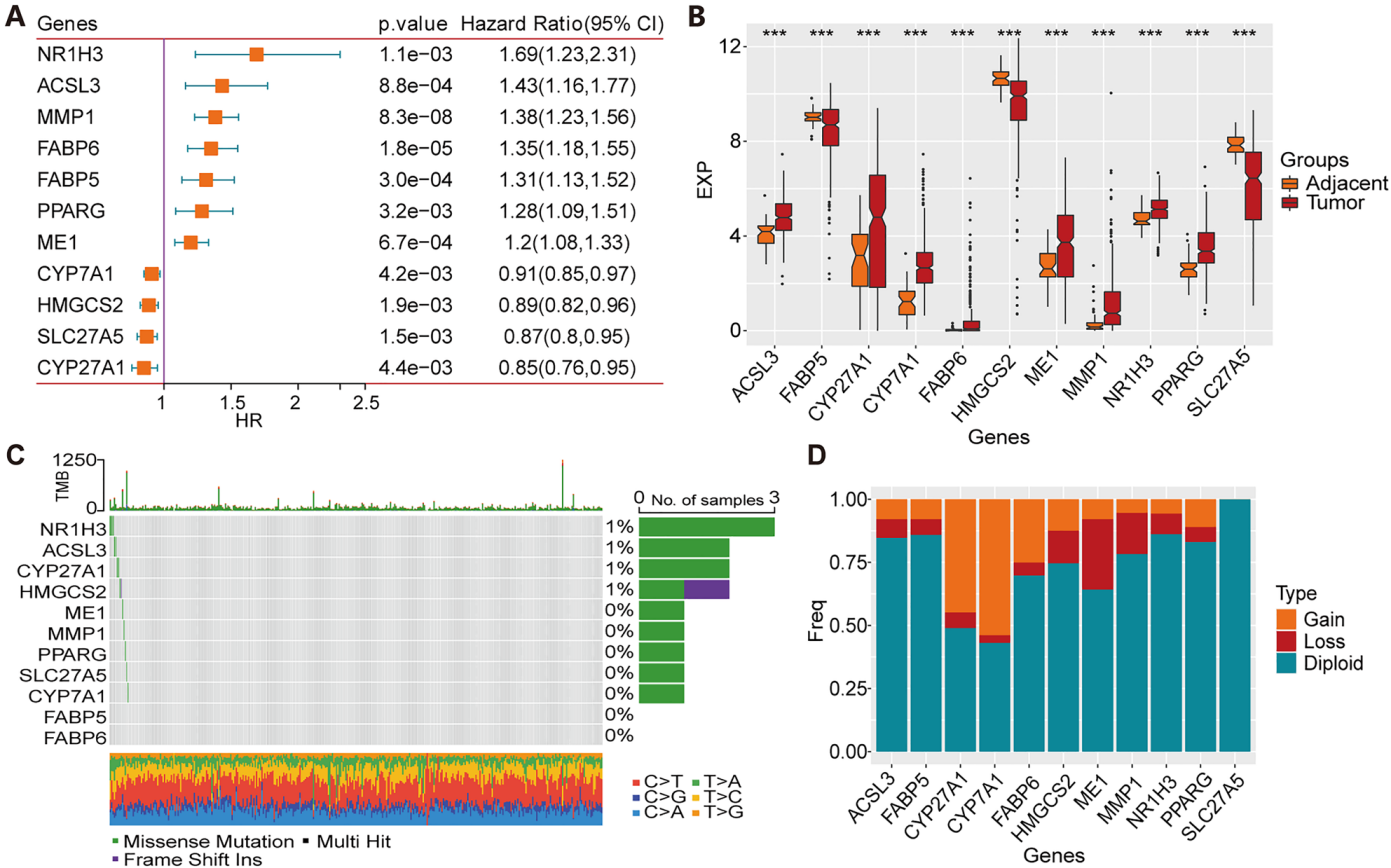
Gene expression and mutation analysis. (**A**) Univariate Cox regression of PPAR signaling pathway-related genes in the TCGA-LIHC dataset. (**B**) Differential gene expression of potential prognosticative genes in HCC tissues and adjacent non-tumour tissues. (**C**) Mutation analysis. (**D**) The frequency of CNV mutations. ***, *p* < 0.001.

A bioinformatics analysis was conducted on the publicly available datasets using the 69 genes associated with the PPAR signaling pathway obtained from the Molecular Signatures Database. To examine the interrelationship between each PPAR-related gene and the prognosis of patients with HCC in the The Cancer Genome Atlas-Liver Hepatocellular Carcinoma (TCGA-LIHC) cohort, the 69 genes were analysed using univariate Cox analysis. Seven genes, namely *NR1H3, ACSL3, MMP1, FABP6, FABP5, PPARG* and *ME1,* were identified as risk factors for survival, while four genes, namely *CYP7A1, HMGCS2, SLC27A5* and *CYP27A1,* were identified as protective factors (Fig. 1A). The gene expression differences of these 11 prognostically relevant genes between HCC tissues and adjacent tissues were further evaluated. Among them, *ACSL3, CYP7A1, CYP27A1, FABP6, ME1, MMP1, NR1H3* and *PPARG* were highly expressed in HCC tissues, while *FABP5, HMGCS2* and *SLC27A5* were highly expressed in adjacent tissues (Fig. 1B). Moreover, the mutation frequency of these 11 genes in HCC was low (≤ 1%) (Fig. 1C). Gene copy number variation (CNV) analysis revealed that most genes had a lower proportion of “loss” compared to “gain” (Fig. 1D).

### Identification of PPAR signaling pathway-related molecular subtypes

**Figure 2:**
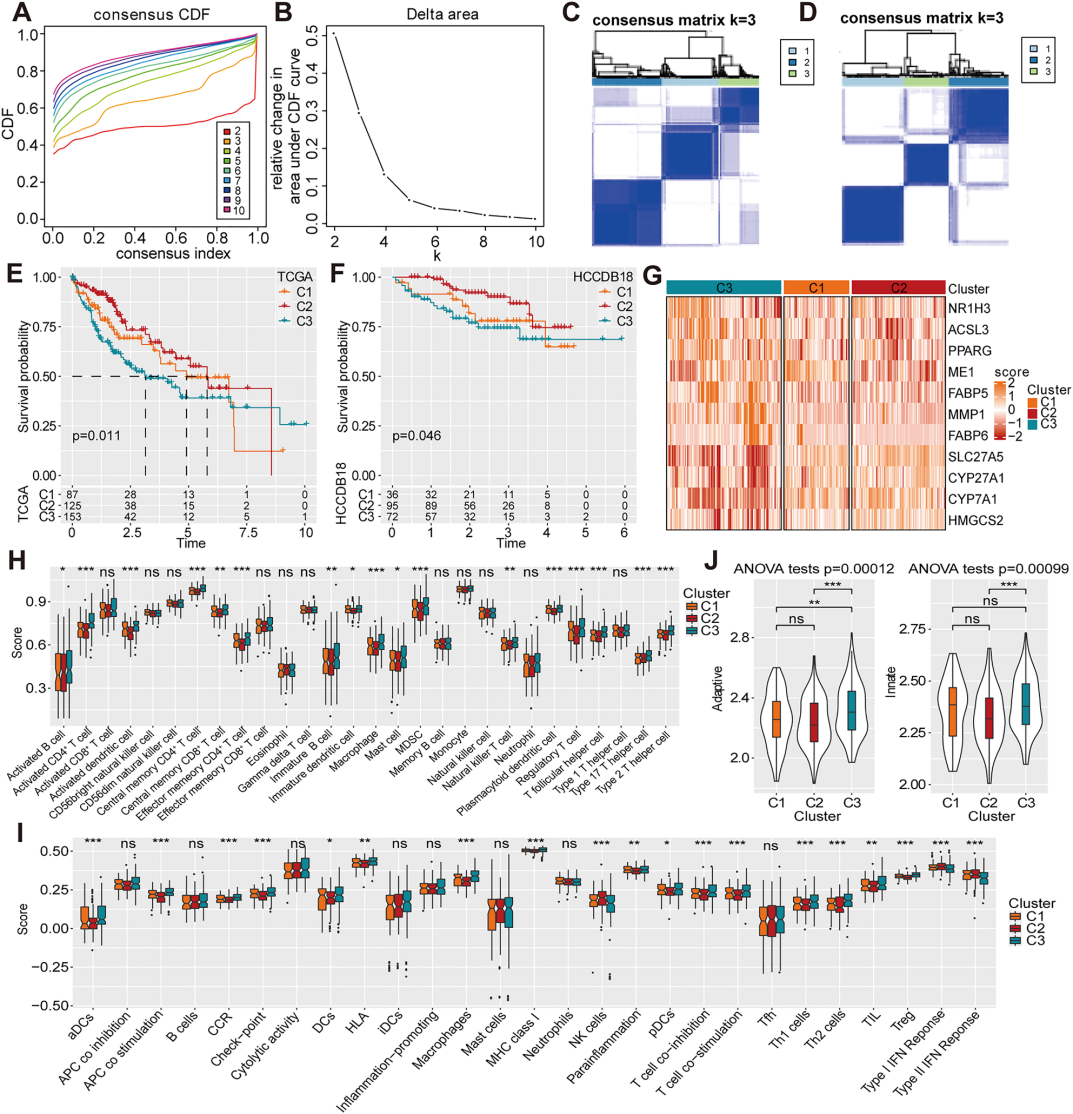
Establishment of PPAR signaling pathway-related molecular subtypes. (**A**) The curve of the cumulative distribution function (CDF). (**B**) Delta area curve of consensus clustering. (**C-D**) Clustering heatmap of samples when consensus matrix k was 3 in the TCGA-Liver Hepatocellular Carcinoma (TCGA-LIHC) and HCCDB18 cohorts. (**E–F**) Kaplan–Meier (K-M) survival analysis in the (**E**) TCGA-LIHC and (**F**) HCCDB18 cohorts. (**G**) Expression heatmap of PPAR signaling pathway-related genes in the TCGA-LIHC cohort. (**H**) Comparison of 28 immune cells evaluated using ssGSEA. (**I**) Comparison of 27 immune components. (**J**) Adaptive immunity and innate immunity scores. ns, non-significant; *, *p* < 0.05; **, *p* < 0.01; ***, *p* < 0.001;***, *p* < 0.001.

Consensus clustering analysis was performed on the 11 genes related to the PPAR signaling pathway. The result indicated that the clustering was more stable when k = 3 (Fig. 2A, B). Based on the TCGA-LIHC and HCCDB18 datasets, the cluster heatmap demonstrated a clear separation between the samples of the three subtypes (Fig. 2C, D). Furthermore, the K–M method showed that the survival outcomes of the three subtypes were statistically different. The median survival time of C2 was significantly longer than that of C3 in the TCGA-LIHC cohort (Fig. 2E). Similarly, in the HCCDB18 dataset, the overall survival of the C3 subtype was significantly shorter than that of the C2 subtype (Fig. 2F). Additionally, the heatmap of the three clusters illustrating the expression of these 11 genes among the subgroups visually demonstrated significant difference (Fig. 2G). Specifically, *SLC27A5, CYP27A1, CYP7A1* and *HMGCS2* were lowly expressed in the C3 subtype and highly expressed in the C2 subtype.

According to previous studies, the single-sample Gene Set Enrichment Analysis (ssGSEA) algorithm assessed the differences in the immune microenvironment among the three subtypes^20,21^. The C1, C2 and C3 subtypes were revealed to have different degrees of immune cell infiltration, especially CD4^+^ T cells, regulatory T cells, activated dendritic cells, MDSCs, plasmacytoid dendritic cells, type 2 T helper cells and T follicular helper cells (*p* < 0.0001) (Fig. 2H). The C3 subtype with poor prognosis had higher levels of immune cell infiltration and checkpoint expression than the C2 subtype (Fig. 2I). One-way analysis of variance was used to test the immune scores in innate and adaptive immunity, revealing similar results to that of ssGSEA (Fig. 2J). These findings indicate that the expression of PPAR signaling pathway-related genes is associated with the outcomes and immune microenvironment in patients with HCC.

### Identification of prognosis-related differentially expressed genes (DEGs) and the construction of a prognostic prediction model

**Figure 3:**
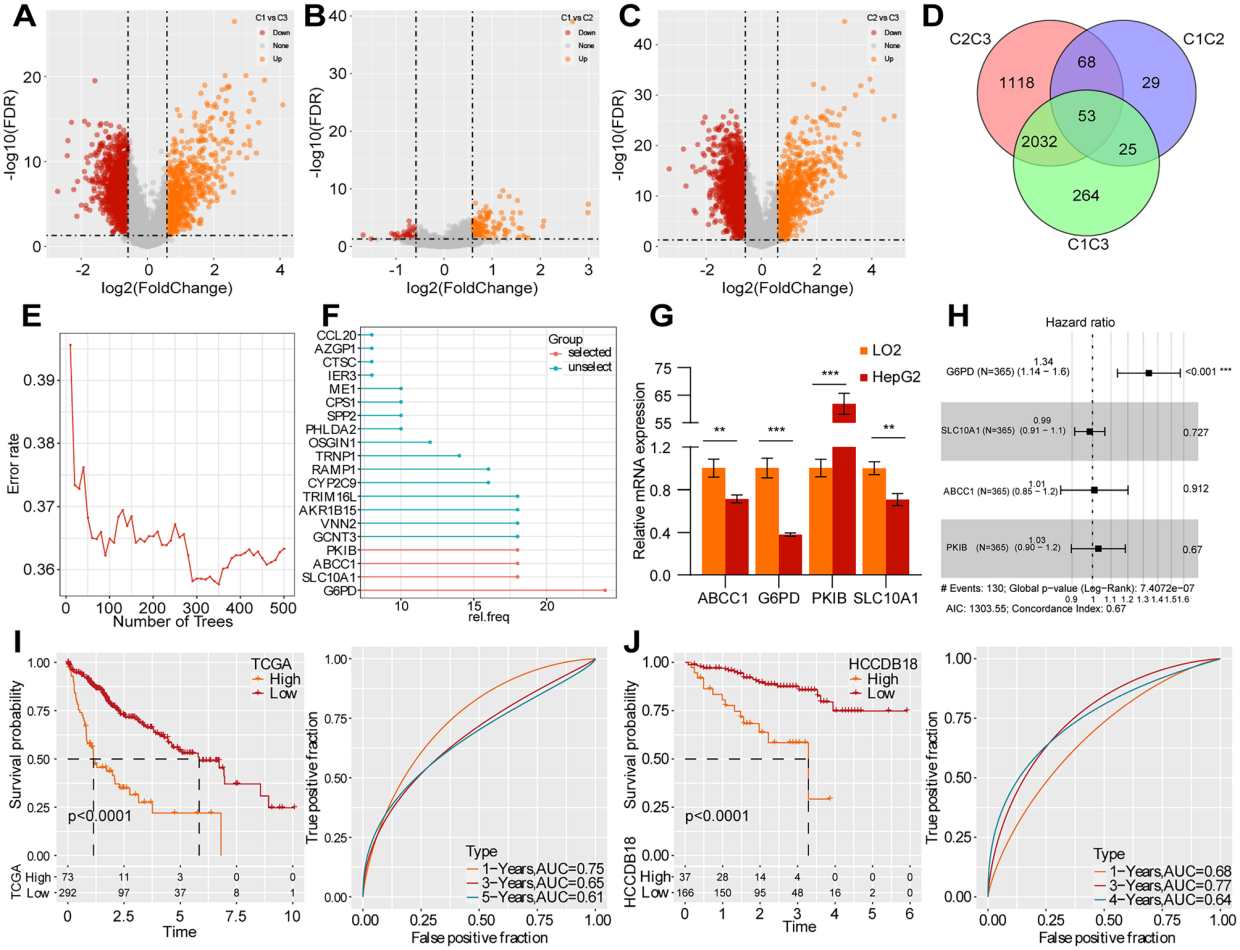
Prognosis-related DEGs and the prognostic prediction model. (**A–C**) Volcanic plot of DEGs between the following pairs of clusters: (**A**) C1 and C3 clusters; (**B**) C1 and C2 clusters; (**C**) C2 and C3 clusters. (**D**) The Venn diagram of DEGs. (**E**) The random survival forest model identifies the top 20 genes with relative importance. (**F**) Using the variable importance method to achieve variable hunting. (**G**) The expression levels of four potential genes in the HepG2 and LO2 cell lines using qRT-PCR. (**H**) Risk coefficients based on multivariate Cox regression analysis. (**I–J**) The K-M survival curves and ROC curves in the (**I**) TCGA-LIHC and (**J**) HCCDB18 cohorts. **, *p* < 0.01; ***, *p* < 0.001.

To further investigate the potential biological behaviour of different molecular subtypes, we identified the DEGs between the following subtypes: C1 and C3, C1 and C2, C2 and C3 in the training dataset (Fig. 3A–C). A total of 53 DEGs were determined among the three subtypes (Fig. 3D). A random survival forest model was constructed using the expression values of the 53 genes in the TCGA-LIHC dataset, and the top 20 genes with relative importance were identified (Fig. 3E). Based on variable importance, four genes were selected, namely *G6PD, SLC10A1, ABCC1* and *PKIB*, for model construction (Fig. 3F). The expression levels of these genes were detected through in vitro experiments on LO2 and HepG2 cells using quantitative reverse transcription-polymerase chain reaction (qRT-PCR), revealing significant different expression levels in HepG2 and normal liver cells (Fig. 3G). Furthermore, a multivariate Cox analysis was performed and the risk coefficient was obtained (Fig. 3H). The predictive model, based on the TCGA-LIHC dataset, consisted of the four genes weighted by their risk coefficients as below: RiskScore = 0.29*G6PD − 0.014*SLC10A1 + 0.01*ABCC1 + 0.03*PKIB. According to RiskScore, the samples were divided into high-risk and low-risk groups, and the predictive ability of the prognostic model was demonstrated in both the training (TCGA-LIHC) and validation (HCCDB18) cohorts (Fig. 3I, J).

### Association of risk score with somatic mutations and tumour mutation burden (TMB)

**Figure 4:**
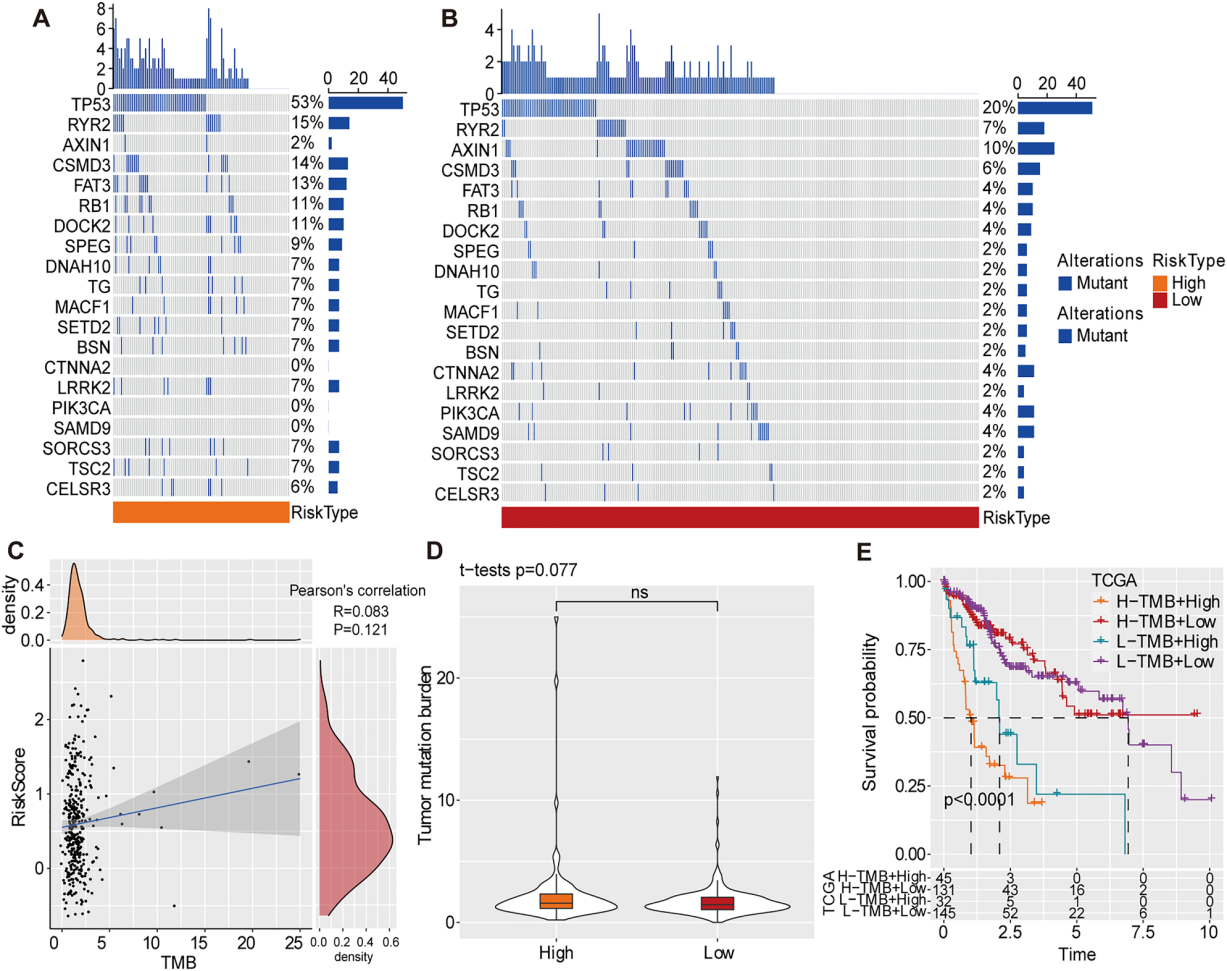
Association of risk score with somatic mutations. (**A–B**) Characteristics of somatic mutation in the (**A**) high-risk group and (**B**) low-risk group. (**C**) Correlation analysis between TMB and risk score. (**D**) The distribution of TMB according to different risk statuses. (**E**) The K-M survival curves of risk groups combined with TMB.

The mutation data of patients with HCC processed by the mutect2 software was downloaded from the TCGA database. We screened 12,704 genes using Fisher’s precision probability test in each group (*p* < 0.05), resulting in 78 genes. The somatic mutation characteristics of the top 20 genes were visualised in a waterfall plot (Fig. 4A, B). *TP53, RYR2, AXIN1, CSMD3, FAT3, RB1, DOCK2, SPEG, DNAH10* and *TG* were the top 10 mutation genes. Patients with a low-risk score had significantly higher frequencies of these mutations, except for *AXIN1*. Typically, patients with high TMB produce more neoantigens and may benefit more from ICI therapy^22^. However, Spearman analysis revealed no significant correlation between TMB and risk score (Fig. 4C), and there was no difference in TMB distribution after risk stratification (Fig. 4D). However, when considering both risk stratification and TMB, the high-TMB + low-risk group and low-TMB + low-risk group exhibited significantly better outcomes compared to the high-TMB + high-risk and low-TMB + high-risk groups (Fig. 4E).

### Correlation between risk score and clinicopathological characteristics

**Figure 5:**
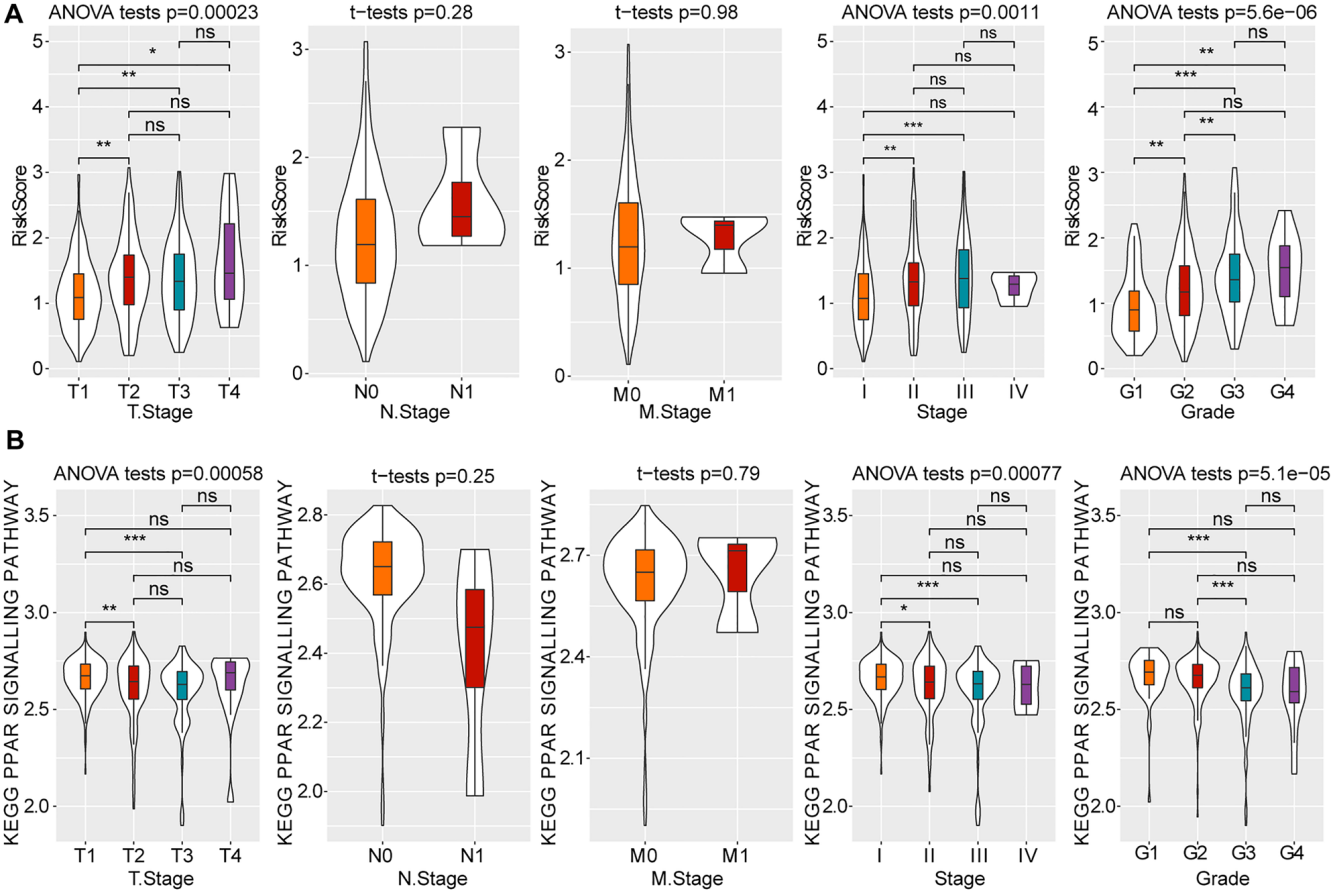
Clinicopathological characteristics based on the TCGA-LIHC dataset. (**A**) The distribution of risk score in different clinicopathological characteristics. (**B**) Evaluation of the PPAR signaling pathway-related gene enrichment score according to different clinicopathological characteristics. ns, non-significant; *, *p* < 0.05; **, *p* < 0.01; ***, *p* < 0.001;***, *p* < 0.001.

We explored the correlation between risk score and each characteristic, including tumour-node-metastasis (TNM) classification, tumour stage and pathology grade. There were significant differences in risk score levels among T stage, stage and grade in the TCGA-LIHC cohort. Patients with HCC with T1, S1, or G1 were significantly associated with lower risk scores. As the clinical grade increased, the risk score also increased (Fig. 5A). Moreover, the analysis of PPAR signaling pathway-related gene enrichment score among T stage, stage and grade revealed similar results as risk score (Fig. 5B).

### Immune and drug sensitivity analysis under different risk states

**Figure 6:**
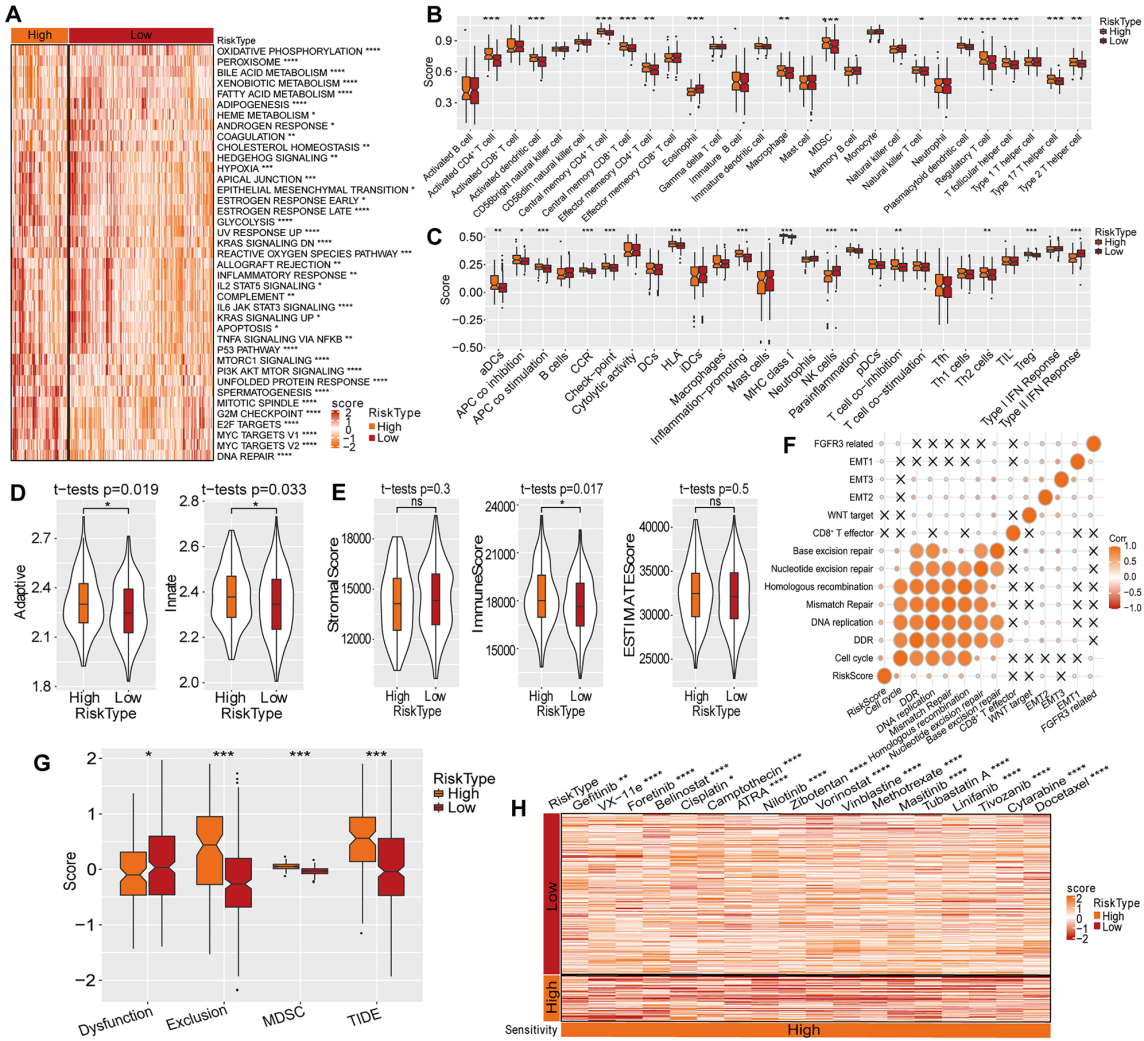
Immune and drug sensitivity based on risk stratification in the TCGA-LIHC dataset. (**A**) Heatmap of pathway scores under different risk statuses. (**B**) Comparison of 28 immune cells. (**C**) Comparison of 27 immune components assessed using ssGSEA. (**D**) Adaptive immunity and innate immunity scores. (**E**) The distribution of ESTIMATE score. (**F**) Analysis of the connection between human-gene signatures score and risk score. (**G**) The TIDE analysis under different risk statuses. (**H**) The sensitivity of patients with HCC to traditional chemotherapeutic drugs. ns, non-significant; *, *p* < 0.05; **, *p* < 0.01; ***, *p* < 0.001;***, *p* < 0.001.

Next, we used ssGSEA method to analyze the enrichment degree of pathways from h.all.v7.4.Symbols.gmt genset, and the heatmap shows 41 pathways with significant differences between high-risk and low-risk groups (Fig. 6A). The analysis of the tumour immune microenvironment (TIME) under different risk states showed variations in the degree of immune cell infiltration. In particular, CD4^+^ T cells, DCs, MDSCs and regulatory T cells were significantly infiltrated in the high-risk samples (Fig. 6B–D). Additionally, we used the Estimation of Stromal and Immune cells in Malignant Tumor tissues using Expression data (ESTIMATE) to evaluate the stromal score, immune score, and ESTIMATE score in the TCGA-LIHC dataset, which revealed that the immune score in the high-risk group was higher than the low-risk group (Fig. 6E). These findings suggested that the overall survival of patients with HCC were related to the abundance of immune components.

The marker genes of 13 pathways were obtained from a previous study^23^, and the risk scores were calculated using the ssGSEA algorithm in the TCGA-LIHC cohort. On evaluating the relationship between risk score and the above pathways, the risk score was observed to be significantly positively correlated with biological processes such as cell cycle, mismatch repair, DNA damage repair, homologous recombination and DNA replication (Fig. 6F).

Additionally, Tumor Immune Dysfunction and Exclusion (TIDE) analysis was conducted to assess the potential prognostic effect of ICI therapy in the defined risk stratification. The TIDE score of the low-risk group in the TCGA-LIHC dataset was lower than the high-risk group, suggesting that patients with HCC in the low-risk group were more likely to benefit from immunotherapy (Fig. 6G). Moreover, the sensitivity of patients with HCC in the high-risk group to 18 traditional chemotherapeutic drugs such as foretinib, belinostat and camptothecin was lower than those in the low-risk group (Fig. 6H).

### Performance of the predictive model in immunotherapy datasets

**Figure 7:**
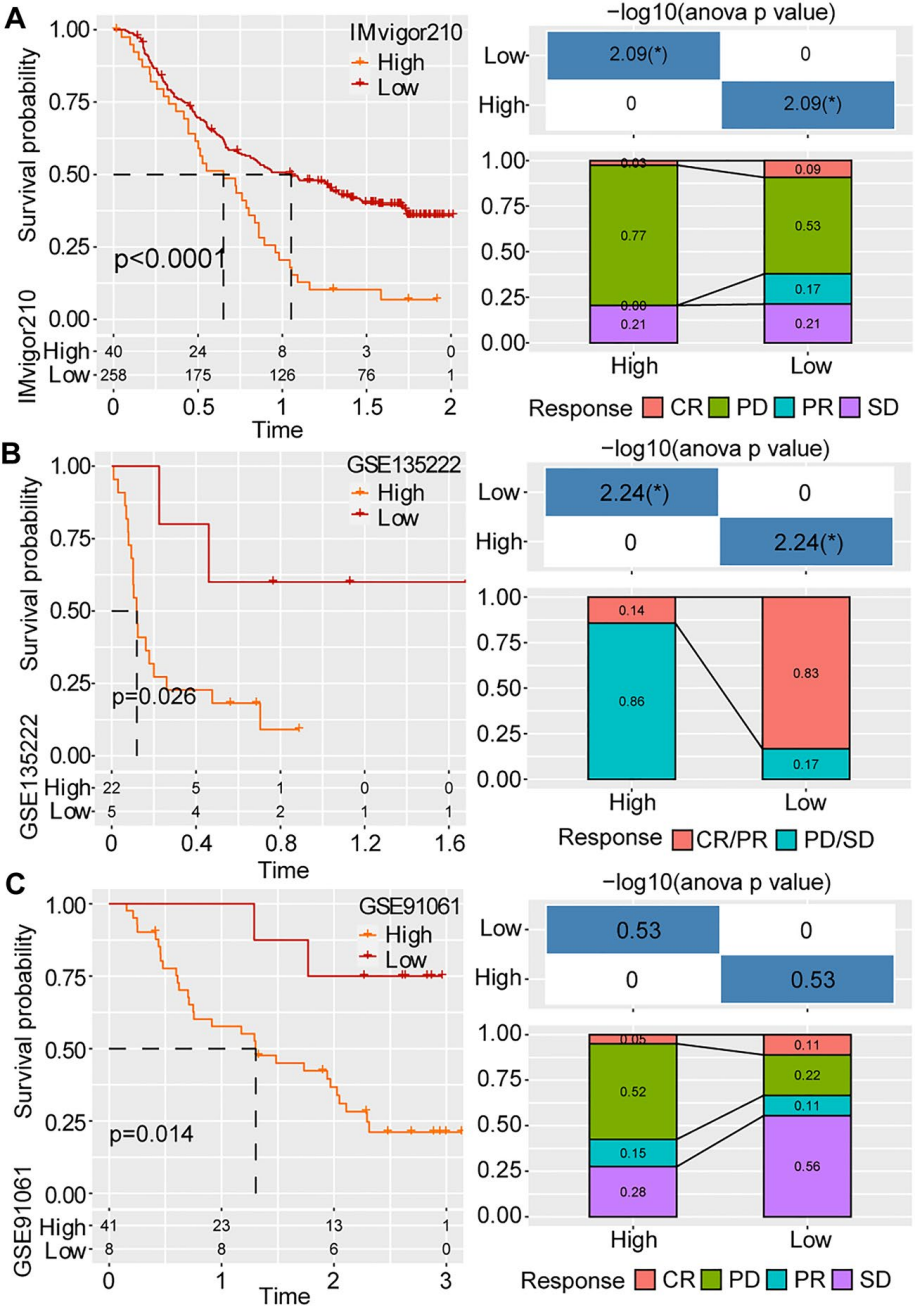
Performance of the prognostic prediction model in immunotherapy dataset. The K-M curves, survival status and disease progression of patients with HCC treated with immunotherapy in the (**A**) IMvigor210, (**B**) GSE135222 and (**C**) GSE91061 datasets.

The new predictive model was used to calculate the risk score of patients with HCC treated with immunotherapy in the IMvigor210 (Fig. 7A), GSE135222 (Fig. 7B) and GSE91061 (Fig. 7C) datasets. The high-risk patients exhibited notably worse overall survival, which verified the robustness of the prognostic model. Additionally, the score showed significantly higher values in patients with progressive disease (PD)/stable disease (SD), which was consistent with the results of the TIDE analysis. Therefore, these findings indicate that the prognostic model can also be applied to predict the response rate of immunotherapy.

### Improvement of the prognostic prediction model combined with clinicopathological features

**Figure 8:**
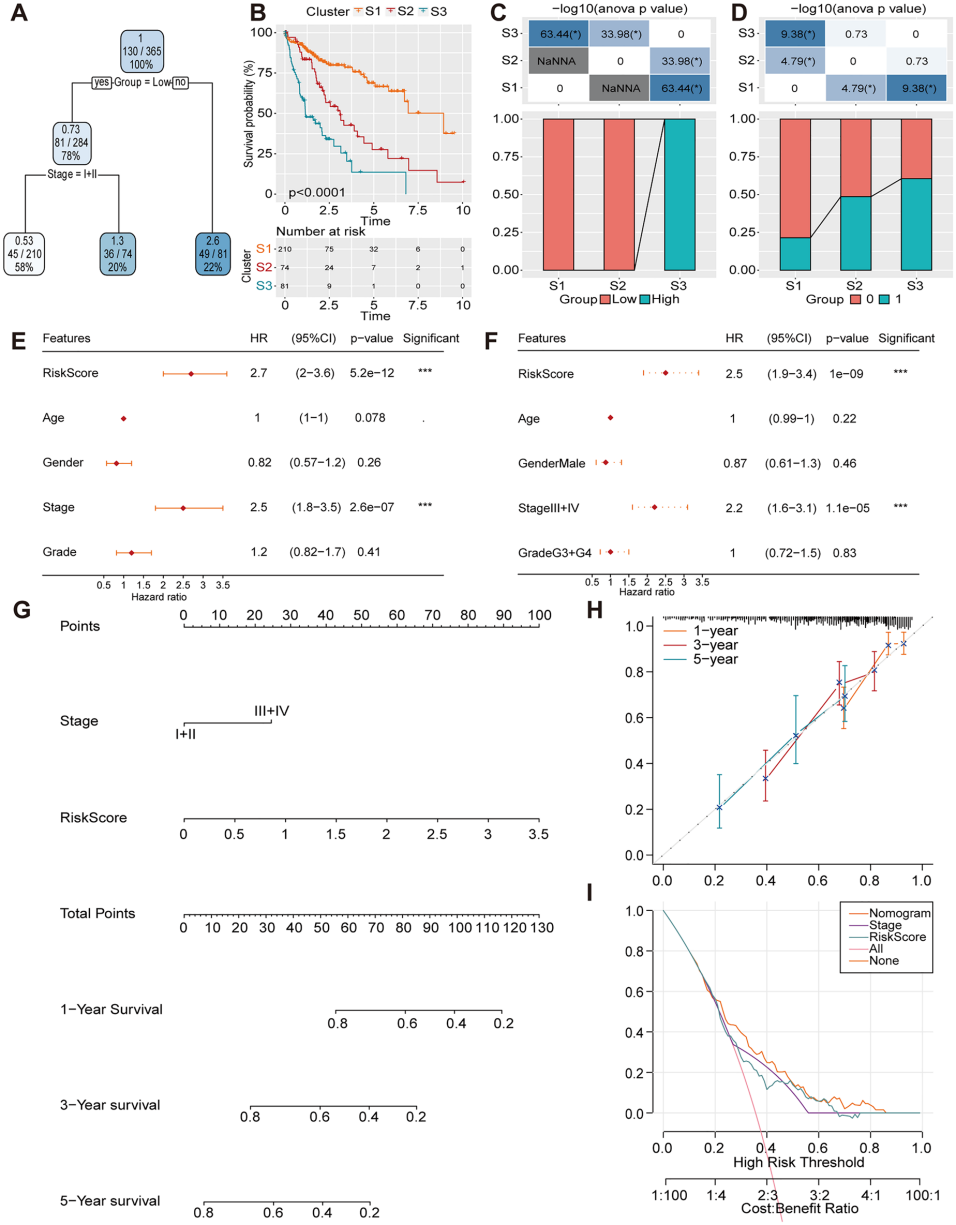
Improvement of the prognostic model. (**A**) The survival decision tree. (**B**) The K-M survival curves. (**C–D**) Comparative analysis in the risk subgroups. (**E**) Univariate Cox regression analysis. (**F**) Multivariate Cox regression analyses. (**G**) The nomogram model. (**H**) The calibration curves and (**I**) the decision curve of the nomogram.

To assist clinical decision-making, we constructed a decision tree based on age, gender, TNM classification, stage, grade and risk score to optimize risk stratification. Based on the two main factors of risk group and T stage, three different risk subgroups were divided (Fig. 8A). There were significant differences in overall survival rates among the subgroups of S1, S2 and S3 (Fig. 8B). Patients with high-risk scores were defined as the S3 group, while patients in the S1 and S2 groups comprised low-risk patients (Fig. 8C). Additionally, distinct survival outcomes were observed among the risk subgroups (Fig. 8D). Univariate and multivariate Cox regression analysis demonstrated that risk score was the most significant factor influencing the prognosis of patients with HCC (Fig. 8E, F). The nomogram results indicated that the risk score had the most significant effect on survival rate prognostication (Fig. 8G). The calibration curve (Fig. 8H) and decision curve (Fig. 8I) were generated to assess the predictive performance of the nomogram and the reliability of the model. These results indicated that, compared to other clinical factors, the risk score and nomogram exhibited the strongest predictive ability for survival.

### Verifying the robustness of the prognostic prediction model through single-cell RNA-sequencing (scRNA-seq) data

**Figure 9:**
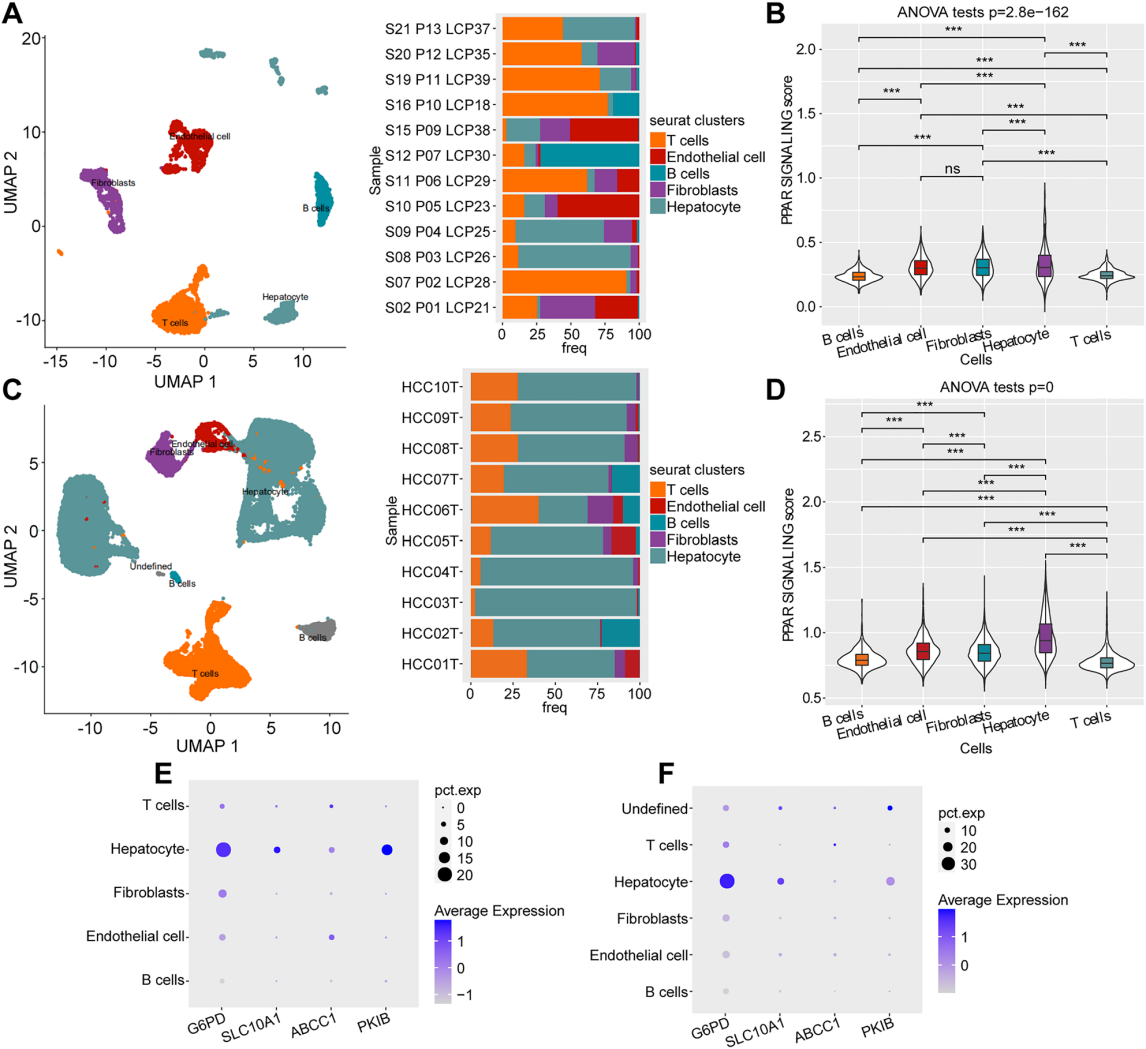
Verification of the robustness of the prognostic prediction model at the single-cell level. (**A, C**) The UMAP plot displayed the proportion of cells in samples of the (**A**) GSE125449 and (**C**) GSE149614 datasets. (**B, D**) The difference of PPAR signaling score in five cell subpopulations in the (**B**) GSE125449 and (**D**) GSE149614 datasets. (**E–F**) The expression of four genes in five cell subpopulations based on the (**E**) GSE125449 and (**F**) GSE149614 datasets. ns, non-significant; ***, *p* < 0.001.

We analysed the scRNA-seq data of HCC samples from the GSE125449 and GSE149614 datasets. Using the cell canonical markers identified previously, all cells were reclassified into T cells, endothelial cells, B cells, fibroblasts and hepatocytes (Fig. 9A, C). On comparing the differences of PPAR signaling pathway-related gene enrichment score in different cell types, it was found that the score values were significantly different among the five cell subpopulations, and the hepatocytes exhibited the highest scores (Fig. 9B, D). Additionally, *G6PD, SLC10A1* and *PKIB* were relatively high-expressed in hepatocytes from both the GSE125449 and the GSE149614 dataset, which indicated that the genes included in the newly constructed prognostic model still had a considerable effect on HCC at the single cell level (Fig. 9E, F).

## Discussion

Despite the implementation of prophylactic vaccination for hepatitis B virus (HBV) and various treatment methods for HCC, the incidence and mortality of HCC remain high due to the large population affected by HBV, alcoholic liver disease, chronic hepatitis C virus infection, NAFLD and autoimmune liver disease. It is therefore urgent to establish an effective prognostic model for estimating the risk of death and making adjuvant treatment decisions in patients with HCC.

In recent years, the relationship between the prognosis of cancer and genes associated with the PPAR signaling pathway have extensively explored. PPAR pathway-related genes have been used to develop predictive models for uterine cervical cancer^24^, renal clear cell carcinoma^25^ and breast cancer^26,27^. The liver is one of the organs with the highest content of PPARα, which is related to the process of energy metabolism and immune regulation. In this study, PPAR pathway-related genes were utilised to establish a model for predicting the prognosis of HCC.

We analysed the potential of 69 genes as prognostic biomarkers and eventually identified 11 PPAR signaling pathway-related genes. Among these genes, the expression of PPARG in HCC and adjacent tissues was significantly different and was identified as a risk factor for disease progression. PPARγ is predominantly expressed in adipose tissue, liver and immune cells. In the liver microenvironment, the balance between PPARγ-mediated inflammatory and anti-inflammatory cytokines influences the hepatic premalignant environment and TME^16,28^. PPARγ signaling also affects the metabolic changes in the HCC-microenvironment, as evidenced by studies showing that inhibiting the PPARγ-ACLY/ACC axis can prevent the de novo synthesis of fatty acids, thereby inhibiting the occurrence and progression of HCC^29^. Furthermore, according to the selected prognosis-related genes, we divided the patient samples obtained from the datasets into three subtypes, identified the DEGs and used four key genes (*G6PD, SLC10A1, ABCC1* and *PKIB*) to establish the new prognostic prediction model.

Metabolic reprogramming, which involves changes in cellular bioenergetics to adapt to hypoxia and a nutrition-deficit environment, plays a crucial role in tumorigenesis^30^. The activation of glucose-6-phosphate dehydrogenase (G6PD), a rate-limiting enzyme in the pentose phosphate pathway, leads to increased NADPH levels, oxidative stress and initiation of carcinogenic signals^31^. A study in 2020 reported that hepatic aldolase B can attenuate the occurrence of HCC by inhibiting G6PD, as demonstrated in Aldob knockout mice^32^. Furthermore, clinical and cellular studies have confirmed that G6PD can activate signal transducer and activator of transcription 3 (STAT3), which is associated with poor outcomes and the migration and invasion of cancer cells^33^. Dingli et al. established a prognostic model of HCC based on endoplasmic reticulum stress-related genes and identified G6PD as a prognostic signature^34^. These findings suggest that G6PD may be a promising prognostic target for HCC.

*SLC10A1*, which encodes a sodium taurocholate co-transporting polypeptide, is not only involved in bile salt-coupled chemotherapeutics transport and aerobic glycolysis but also serves as a receptor for HBV^35–37^. It has been reported that overexpression of SLC10A1 at the cellular level exhibits a significant tumour suppressive effect, inhibiting aerobic glycolysis and HCC proliferation and migration^36^. Two previous researches using glycolysis-related genes and lipid metabolism-related genes revealed a strong prognostic efficacy of SLC10A1 as a marker signature^38,39^. *ABCC1* encodes a transporter associated with multidrug resistance, which has important significance in the treatment and prognosis of HCC^40^. Furthermore, the upregulation of *ABCC1* in HCC has also been associated with poor prognosis^41^. The role of protein kinase inhibitor β (PKIB) as a prognostic marker of HCC was first reported in this study. More mechanism studies are needed to explore the molecular function of PKIB in HCC progression.

To achieve immune escape, the immune cell components of the tumour undergo changes. Although the effect of the TIME, which is composed of different components, on the development, metastasis and recurrence of HCC remains unknown, it has implications for choosing immunotherapy strategies to achieve optimal therapeutic effects^42,43^. In a study based on 919 cases of HCC, the TIME was divided into three subtypes, among which the high-immune subtype with increased B cell and T cell infiltration was associated with a better prognosis^44^. It is generally speculated that CD8^+^ T cells, CD4^+^ T cells, memory T cells, B cells and M1 macrophages are associated with good prognosis, while M2 macrophages, regulatory T cells and regulatory B cells are associated with poor prognosis. With the rapid development of new technologies such as cytometry by time-of-flight (CyTOF) analysis, the understanding of tumor immunity has been deepened and refined. It is worth mentioning that Tregs fully express checkpoint molecules such as cytotoxic T lymphocyte-associated antigen 4 and programmed cell-death 1 receptor and thus become a direct target for ICIs^45^. In our study, samples with high-risk scores had higher levels and broader immune cell enrichment than the low-risk score samples, presumably induced by high TMB. In addition to immune cells, fibroblasts, as the main cell type in cancer-related stroma, participate in tumor-microenvironment interactions by secreting extracellular matrix proteins and growth factors^46,47^. We also looked at this subpopulation in scRNA-seq analysis and found that the PPAR signaling pathway-related gene enrichment score was higher in fibroblasts than in T cells and B cells, which may help to explore potential interaction processes.

In this study, a new prediction model for HCC was established, comprising four genes based on PPAR signaling pathway. The multi-level, multi-dimensional and multi-database verification showed that the model had good performance. However, this study has several limitations. Animal experiments or prospective clinical studies have not been conducted to validate the prognostic prediction model in real-world settings. There may be some informatics bias in the samples obtained from public databases. Further testing, evaluation and application of the prognostic prediction model to address these limitations will be the emphases of our future research.

## Conclusions

In conclusion, we systematically elucidated the prognostic value of PPAR signaling pathway-related genes in patients with HCC and established a prognostic model comprising a four-gene signature (G6PD, SLC10A1, ABCC1 and PKIB). These gene signatures, which serve as potential biomarkers, are closely related to the survival of patients with HCC, thereby aiding in the personalised management of HCC.

## Materials and methods

### Data sources

The gene expression and corresponding clinical data of patients with HCC in the training dataset were obtained from the publicly available database TCGA-LIHC. The expression profile of the HCCDB18 dataset, which serves as the validation cohort, was obtained from the Hepatocellular Carcinoma Database^48^. Patients treated with immunotherapy were selected from the IMvigor210 cohort^23^ and Gene Expression Omnibus (GEO) datasets (GSE91061 and GSE135222). Additionally, scRNA-seq data were acquired from GSE125449 and GSE149614 datasets. Moreover, the 69 genes enriched in the Kyoto Encyclopedia of Genes and Genomes (KEGG) ‘PPAR signaling pathway’ were obtained from the Molecular Signatures Database (Table S1)^49^.

### Cell culture and qRT-PCR method

The hepatoma cell line HepG2 and normal hepatocyte line LO2 were purchased from the Chinese Academy of Sciences (Shanghai, China). Dulbecco’s modified Eagle’s medium (Gibco, USA) and RPMI-1640 medium (Gibco, USA) were used to culture HepG2 and LO2 cells, respectively. All culture systems contained 10% fetal bovine serum (Gibco, USA) and 1% penicillin/streptomycin (Beyotime, China) in a 5% CO2 incubator at 37 °C.

Total RNA was extracted from cultured cells using the RNeasy Mini Kit (QIAGEN, USA). Besides, the PrimeScript RT reagent Kit (Takara, Japan) was used to reverse RNA to cDNA and TB Green Premix (Takara, Japan) was utilized to amplify DNA. Table S2 shows the primer sequences (Tsingke Biotech, China) used for qRT-PCR. The 2^-ΔΔCt^ method was carried out to the relative quantification of the target genes compared to the reference gene GAPDH^50^.

### Identification of molecular subtypes in the training dataset

Firstly, the ConsensusClusterPlus package (specific parameters: clusterAlg = ‘pam’, distance = ‘spearman’, 500 sampling repetitions and a sampling ratio of 0.8) was used to determine the molecular subtypes based on PPAR signaling pathway-related genes. Cumulative distribution function was adopted to determine the optimal number of clusters and principal component analysis (PCA) were performed to observe the separation between different subtypes.

### DEGs screening and construction and validation of a PPAR signaling pathway-related model

To identify differentially expressed PPAR signaling pathway-related genes, limma package was utilised to analyse the DEGs in the TCGA-LIHC dataset (FDR < 0.05, |log2FC|> log2(1.5)). The Random Survival Forest algorithm was employed to compress the 53 DEGs in the TCGA-LIHC dataset using the randomForestSRC package. Key prognostic genes with relative importance were identified using the variable importance method of variable hunting. Additionally, multivariate Cox analysis was performed using the selected four genes to obtain the hazard ratio and construct the optimal regression model.

Based on the risk coefficient of each gene, the following equation was developed to estimate the outcome of patients with HCC: risk score = Σβi × Expi, where i represents the prognostic gene, β represents the Cox regression coefficient and Exp indicates the normalised mRNA expression level^51,52^.

The new score for each patient with HCC in the training and validation cohorts was categorised at the optimal cutoff point (high risk or low risk) using the surv_cutpoint function in survminer package. Kaplan–Meier survival analysis was used to compare the differences in median survival time and overall survival. Furthermore, the receiver operating characteristic (ROC) curve analysis was performed using the timeROC package in R software.

### Somatic mutation analysis

The ‘maftools’ R package was used to analyse and visualise the mutation frequency. Tumour mutation burden was computed to assess the response to immunotherapy of patients with HCC.

### Immune feature estimation

ssGSEA was employed to calculate PPAR signaling pathway-related gene enrichment score, immune scores, pathway scores and the relative abundance of immune cells infiltration in tissues. Additionally, the ESTIMATE algorithm was used to evaluate the stromal score, immune score and ESTIMATE score in the TCGA-LIHC dataset^53^. TIDE algorithm was also used to evaluate the potential clinical efficacy of ICIs^54^.

### Clinical prediction and decision-making

We evaluated the sensitivity of conventional chemotherapy drugs using the pRRophetic package. To further improve the prognostic model, we established a decision tree and a nomogram. The calibration curve of the nomogram was generated to examine the predictive value among the predicted 1-, 3- and 5-year survival rates and the standard curve. The decision curve was plotted to evaluate the reliability of the prognostic prediction model.

### scRNA-seq data processing

The scRNA-seq data was processed as follows: (1) scRNA-seq data were filtered (each gene was expressed in not less than three cells and each cell expressed at least 200 genes). (2) The percentage of mitochondria and rRNA was calculated to exclude low-quality cells, and the genes expressed in each cell were confirmed to be between 200 and 8000, the mitochondrial content was below 10%, and the unique molecular identifier of each cell was not less than greater than 200. (3) The sample data was normalised using log-normalization. (4) The highly variable genes were filtered using the FindVariableFeatures function. (5) Genes were scaled using the ScaleData function, and the PCA was performed to reduce the dimension to identify anchor points. (6) To cluster the cell, a selection of dim = 10 was performed using the FindNeighbors and FindClusters functions (Resolution = 0.05). Cell markers were obtained from previous studies^55–57^, and the data were reclassified into five types of cells based on the expression of these marker signatures.

### Statistical analysis

All statistical data were analysed using R (version 4.0). All *p*-values were two-sided and statistical significance was set at *p* < 0.05.

### Supplementary Information

Supplementary Tables.

## Data Availability

Publicly accessible datasets were analyzed in this study. The data can be found in the TCGA (http://www.cancer.gov/tcga), HCCDB (http://lifeome.net/database/hccdb), and GEO (https://www.ncbi.nlm.nih.gov/geo). According to the journal’s guidelines, the data involved in this study are available from the corresponding author on request.
