# Supplementary material for: Development of a promising PPAR signaling pathway-related prognostic prediction model for hepatocellular carcinoma
Source: Sci Rep. 2024 Feb 28;14:4926. doi: 10.1038/s41598-024-55086-6 (PMC10902383; doi:10.1038/s41598-024-55086-6)
Supplement: Supplementary file 1 — Supplementary Tables. [file 41598_2024_55086_MOESM1_ESM.pdf]

Supplementary material

Development of a Promising PPAR Signaling Pathway-Related Prognostic Prediction Model for Hepatocellular Carcinoma

Qingmiao Shi[1#], Yifan Zeng[1#], Chen Xue[1], Qingfei Chu[1], Xin Yuan[1], Lanjuan Li[1*]

[1]State Key Laboratory for Diagnosis and Treatment of Infectious Diseases, National Clinical Research Center for Infectious Diseases, National Medical Center for Infectious Diseases, Collaborative Innovation Center for Diagnosis and Treatment of Infectious Diseases, The First Affiliated Hospital, Zhejiang University School of Medicine, Hangzhou 310003, China

[#]These authors contributed equally to this work.

***Corresponding author: Lanjuan Li**

State Key Laboratory for the Diagnosis and Treatment of Infectious Diseases, The First Affiliated Hospital, Zhejiang University School of Medicine, 79 Qingchun Rd., Hangzhou City 310003, China. Tel: 86-571-87236458; Fax: 86-571-87236459

E-mail: ljli@zju.edu.cn

Supplementary Table1. The gene set of 69 PPAR signaling pathway-related genes

No.	Gene	No.	Gene	No.	Gene
1	ACAA1	24	CYP27A1	47	OLR1
2	ACADL	25	CYP4A11	48	PCK1
3	ACADM	26	CYP4A22	49	PCK2
4	ACOX1	27	CYP7A1	50	PDPK1
5	ACOX2	28	CYP8B1	51	PLIN1
6	ACOX3	29	DBI	52	PLTP
7	ACSL1	30	EHHADH	53	PPARA
8	ACSL3	31	FABP1	54	PPARD
9	ACSL4	32	FABP2	55	PPARG
10	ACSL5	33	FABP3	56	RXRA
11	ACSL6	34	FABP4	57	RXRB
12	ADIPOQ	35	FABP5	58	RXRG
13	ANGPTL4	36	FABP6	59	SCD
14	APOA1	37	FABP7	60	SCD5
15	APOA2	38	FADS2	61	SCP2
16	APOA5	39	GK	62	SLC27A1
17	APOC3	40	GK2	63	SLC27A2
18	AQP7	41	HMGCS2	64	SLC27A4
19	CD36	42	ILK	65	SLC27A5
20	CPT1A	43	LPL	66	SLC27A6
21	CPT1B	44	ME1	67	SORBS1
22	CPT1C	45	MMP1	68	UBC
23	CPT2	46	NR1H3	69	UCP1

Note: The gene set was downloaded from the following site: http://www.gsea-msigdb.org/gsea/msigdb/human/annotate.jsp?geneSetName=KEGG_PPAR_SIGNALING_PATHWAY

Supplementary Table2. The information of primers sequences for qRT-PCR.

Primer name	Sequence (5'-3')	Base
G6PD-F	CGGCGACATCTTCCACCAG	19
G6PD-R	TCAGGGAGCTTCACGTTCTTGT	22
SLC10A1-F	TGGGAAATGGCACCTACAAA	20
SLC10A1-R	TACTGGAAATGCTGGAGAAAGA	22
ABCC1-F	CCACAACAGCACCGCAGAAC	20
ABCC1-R	ATGTAGTCCCAGTACACGGAAAGC	24
PKIB-F	GACATTTAATCTGGTGGTAACTGTGG	26
PKIB-R	TCCTTCAGTGCGATTTGGAAGT	22
GAPDH-F	ACATCAAGAAGGTGGTGAAGCAG	23
GAPDH-R	GTCAAAGGTGGAGGAGTGGGT	21
